# Potential Research Tool of Stem Cells from Human Exfoliated Deciduous Teeth: Lentiviral Bmi-1 Immortalization with EGFP Marker

**DOI:** 10.1155/2019/3526409

**Published:** 2019-03-10

**Authors:** Siqi Yao, Lingping Tan, Huan Chen, Xiaojun Huang, Wei Zhao, Yan Wang

**Affiliations:** Guanghua School of Stomatology, Hospital of Stomatology, Sun Yat-sen University, Guangdong Provincial Key Laboratory of Stomatology, 56 Lingyuanxi Road, Guangzhou 510055, China

## Abstract

Stem cells from human exfoliated deciduous teeth (SHED) are a favourable source for tissue engineering, for its great proliferative capacity and the ease of collection. However, the transplantation of stem cells and the study of stem cell-based tissue engineering require massive stem cells. After long-term expansion, stem cells face many challenges, including limited lifespan, senescence, and loss of stemness. Therefore, a cell line capable of overcoming those problems should be built. In this study, we generated a Bmi-1-immortalized SHED cell line with an enhanced green fluorescent protein (EGFP) marker (SHED-Bmi1-EGFP) using lentiviral transduction. We compared this cell line with the original SHED for cell morphology under a microscope. The expression of Bmi-1 was detected with Western blot. Replicative lifespan determination and colony-forming efficiency assessment were using to assay proliferation capability. Senescence-associated *β*-galactosidase assay was performed to assay the senescence level of cells. Moreover, multipotency, karyotype, and tumour formation in nude mice of SHED and SHED-Bmi1-EGFP were also tested. Our results confirmed that Bmi-1 immortalization did not affect the main features of SHED. SHED-Bmi1-EGFP could be passaged for a long time and stably expressed EGFP. SHED-Bmi1-EGFP at a late passage showed low activity of *β*-galactosidase and similar multilineage differentiation as SHED at an early passage. The immortalized cells had no potential tumourigenicity ability *in vivo*. Moreover, we provided some suggestions for potential applications of the immortalized SHED cell line with the EGFP marker. Thus, the immortalized cell line we built can be used as a functional tool in the lab for long-term studies of SHED and stem cell-based regeneration.

## 1. Introduction

Teeth and their surrounding tissues must be repaired or regenerated after traumatic injuries, caries lesions, and periodontal diseases [1]. Because of the restricted self-healing capability of the human body, these pathologies have been mainly clinical challenges. Stem cell-based tissue engineering has become an unstoppable and promising technology to achieve tooth repair and regeneration [2–5]. After injury or inflammation, dental stem cells near the nidus can be attracted by released molecules and can create a new generation of functioning cells to repair dental tissue [6]. However, stem cells in situ are usually inadequate for the whole regeneration. This process necessitates the ex vivo expansion of stem cells isolated from specific tissues. At the same time, many studies on stem cell-based tissue engineering require a large number of stem cells. The limited lifespan, senescence, and loss of stemness after long-term culture have become the bottleneck in tissue engineering development [7–9].

Among multifarious dental stem cells, stem cells from human exfoliated deciduous teeth (SHED) have drawn much attention over the years [10, 11]. These cells are derived from the deciduous dental pulp, proliferate rapidly, and can differentiate into osteogenic, adipogenic, odontogenic, chondrogenic, and neurogenic cells *in vitro* [12]. These cells also enhance the *in vivo* effect of bone formation and wound healing [13]. The final destiny of deciduous teeth is either shedding naturally or being extracted under certain conditions. Obtaining deciduous dental pulp stem cells can be achieved with little or no trauma. SHED showed greater proliferation capacity than adult dental pulp stem cells (DPSC) [14–16] and other origins of adult stem cells [17–19]. These advantages make SHED a promising source of seed cells for tissue engineering.

Diverse methods were used to achieve cell immortalization. By expressing genes like simian virus-40 large T-antigen (SV-40LT), Stat3, and TERT, several types of human cells were immortalized [20–28]. Bmi-1 is a polycomb group gene that can suppress the transcription of p16^Ink4*α*^ and p19^Arf^ [29]. Immortalized human cell lines can also be generated by the overexpression of Bmi-1 [30–32]. Besides, it has been reported that Bmi-1 is able to regulate cell proliferation, apoptosis, and differentiation of human mesenchymal stem cells (hMSCs). Overexpression of Bmi-1 in hMSCs reduces apoptosis and increased cell proliferation by repressing p16 (INK4A) [33]. Bmi-1 inhibits senescence and enhances the immunomodulatory properties of hMSCs [34]. There is a correlation between Bmi-1 and cancer stem cell-like properties [35–37].

In this study, we hypothesized that Bmi-1 can lead to the immortalization of SHED without affecting its main features, and we generated an immortalized SHED cell line with an EGFP marker. The resulting cells were compared to the original SHED for cell morphology, senescence level, proliferation capability, multipotency, and karyotype. We confirmed that the cells had no potential tumourigenicity *in vivo*. Additionally, we marked the immortalized cells with enhanced green fluorescent protein (EGFP) and provided some suggestions for potential applications. The immortalized cell line we built can be used as a functional tool in the lab for long-term studies on SHED and stem cell-based regeneration.

## 2. Materials and Methods

### 2.1. Isolation of SHED and Cell Culture

Healthy deciduous teeth were extracted from 12 children (7-8 years old, 6 male and 6 female) because of retained deciduous teeth at the Paediatric Dentistry Department, the Affiliated Stomatological Hospital of Sun Yat-sen University. Each patient's legal guardians signed informed consent. Ethics committee approval was granted by the Affiliated Stomatological Hospital of Sun Yat-sen University. The deciduous teeth used in our study were near natural exfoliation, with more than two-thirds of the root absorption and without any caries, restoration, periapical lesions, or internal resorption. SHED were collected and cultured as previously reported [12]. Briefly, we washed the surface of each tooth with sterile phosphate-buffered solution (PBS), split the tooth around the cementum-enamel junction, and removed the pulp. The pulp tissues were cut into 1 mm^3^ pieces and were digested with 3 mg/mL collagenase type I and 4 mg/mL dispase (Gibco-BRL, Grand Island, NY, USA) for 1 hour at 37°C. A single cell was suspended in complete DMEM (Gibco, Grand Island, NY, USA) containing 10% (*v*/*v*) foetal bovine serum (Gibco), 100 U/mL penicillin, 100 *μ*g/mL streptomycin (HyClone, Logan, UT, USA), and 5 mM L-glutamine (Gibco) at 37°C in 5% CO_2_. The culture medium was changed every 3 days. After reaching 80%, the SHED were subcultured. The SHED used in this study were a mixture of cells collected from 12 children to decrease individual variation.

### 2.2. Establishment of a Stable Cell Line

The EGFP gene was produced with PCR and was inserted into the pMSCV plasmid (provided by Dr. Yan Yuan, University of Pennsylvania) between the BglII and XhoI sites. The recombined plasmid was transformed into DH5*α E. coli*, followed by screening the transformants for valid insertion of the EGFP fragment with restriction and sequencing analyses. To produce the lentivirus, 4.5 *μ*g pMSCV-EGFP, 4.5 *μ*g pFIV-34N lentiviral gag-pol packaging vector, and 0.57 *μ*g pVSV-G envelope vector were cotransfected into HEK-293T cells with the calcium phosphate transfection method. Then, 72 hours later, lentiviral particles were harvested and concentrated. The SHED were infected with lentivirus at 2,500 rpm for 1 h at room temperature with 8 *μ*g/mL polybrene and were incubated at 37°C in 5% CO_2_ for 4 h. The inocula were replaced with fresh media. At 48 hours, successfully infected cells were selected using puromycin treatment. Another plasmid, pMSCV-Bmi-1, was provided by Mu-Sheng Zeng, State Key Laboratory of Oncology in Southern China, and Department of Experimental Research. The process of lentiviral production was similar to the process described above. We used the Bmi-1 lentivirus to infect SHED that successfully expressed EGFP. After selection, the successfully infected cells were renamed SHED-Bmi1-EGFP.

### 2.3. Quantitative Real-Time Reverse Transcription Polymerase Chain Reaction (qRT-PCR)

The total RNA was extracted from cells using a TRIzol reagent (Life Technologies), and DNA contamination was removed using RNase-free DNase I (Takara, Shiga, Japan). The Reverse Transcriptase M-MLV Kit (Takara, Shiga, Japan) was used to synthesize first-strand cDNA. Gene expression levels were quantified with qRT-PCR and a SYBR Green kit (Roche, Basel, Switzerland) in combination with gene-specific primers. The GAPDH gene was used to normalize the mRNA data. The gene-specific primers used are listed in Table 1.

### 2.4. Western Blot

The cells were lysed in RIPA lysis buffer (50 mM Tris-HCl pH 7.5, 150 mM NaCl, 1% NP-40, 0.5% sodium deoxycholate, and 0.1% SDS with complete protease inhibitor cocktail). The protein concentration was evaluated using the BCA protein assay kit (Pierce). Protein (40 *μ*g) from each cell extract was separated with electrophoresis on sodium dodecyl sulphate polyacrylamide gels and was transferred to a nitrocellulose membrane. Next, the membranes were blocked for 1 hour at room temperature with 5% nonfat milk and were incubated at 4°C overnight with diluted primary antibodies against Bmi-1 (Cell Signaling Technology) and *β*-actin (Sigma-Aldrich, St. Louis, MO, USA). The membranes were finally incubated with secondary antibodies for 1 hour at room temperature and were analysed using an Odyssey 2-colour infrared laser imaging system (LI-COR Biosciences, Lincoln, NE, USA). The relative intensity of labelled protein bands was quantified using Image-Pro Plus 5.0 software (Media Cybernetics Inc., Rockville, MD, USA).

### 2.5. Replicative Lifespan Determination

The cells were seeded at 1 × 10^3^ cells/T25. After 3 days, the cell number was counted, and the same number of cells was passaged and cultured. Population doubling was calculated as log2 (cell number at subculture).

### 2.6. Senescence-Associated *β*-Galactosidase Assay

The cells cultured in 6-well dishes were washed with PBS and fixed with fixation buffer for 15 minutes at room temperature. We stained the cells overnight at 37°C with a senescence-associated *β*-galactosidase assay working reagent that was prepared according to the specifications of the Senescence *β*-Galactosidase Staining Kit (Beyotime Biotech, Shanghai, China). The cells were viewed under a light microscope, and those stained with deep blue were counted as senescent.

### 2.7. Colony-Forming Efficiency Assessment

To evaluate the colony-forming efficiency, the cells were seeded in 10 cm dishes at a density of 1 × 10^3^ cells per dish. After culturing for 12 days, the cells were washed with PBS, fixed with fixation buffer, and stained with 0.1% (*w*/*v*) crystal violet (Sigma-Aldrich). Aggregates of 50 or more cells were counted as a colony unit.

### 2.8. Multilineage Differentiation Assays

The cells were seeded in 12-well dishes at a density of 5 × 10^4^ cells/well. After the cells grew to 80% confluence, the culture medium was replaced with a differentiation culture medium. For osteogenic differentiation, the medium used was DMEM containing 10% foetal bovine serum, 10 mM *β*-glycerophosphate, 10 nM dexamethasone, and 50 *μ*g/mL ascorbic acid. Then, 14 days later, the cells were fixed and stained following the manufacturer's instructions for the BCIP/NBT alkaline phosphatase colour development kit (Beyotime Biotech). ALP activity was measured following the manufacturer's instructions for the Alkaline Phosphatase Assay kit (Beyotime Biotech). After 14 days of osteogenic induction, the cells were fixed with 4% paraformaldehyde and stained with Alizarin Red. The red positions were recognized as mineralized nodules. After images were recorded, nodules were dissolved using 10% (*w*/*v*) cetylpyridinium chloride (Sigma-Aldrich), and absorbance was evaluated spectrophotometrically at 562 nm.

For adipocyte differentiation, the differentiation induction medium contained 1 M dexamethasone, 0.2 mM indomethacin, 0.1 mg/mL insulin, and 1 mM 3-isobutyl-1-methylxanthin (Sigma-Aldrich), and the differentiation maintenance medium consisted of 0.1 mg/mL insulin in standard medium. The cells were cultured in induction medium for 3 days and in maintenance medium for 1 day. The cells were later switched to induction medium again. After 21 days, the cells were fixed with 4% paraformaldehyde and stained with Oil Red O.

### 2.9. Cytogenetic Analysis

The cytogenetic analysis of cells was performed in collaboration with NuwaCell Ltd. (http://www.nuwacell.com). Briefly, the cells were cultured in T25 flasks until 80% confluence. The cells were treated with 50 ng/mL colcemid solution for 2 hours at 37°C. Metaphase cells were analysed using standard Giemsa staining (G-banding). A total of 5 metaphase cells in each group were randomly selected to analyse genomic stability.

### 2.10. Tumourigenicity in Nude Mice

The animal experiments were conducted in compliance with Sun Yat-sen University animal care and with committee approval. A total of 20 nude mice at 6–8 weeks of age were divided into four groups: SHED-ori P4, SHED-Bmi1-EGFP P40, CAL-27 (human tongue squamous cell carcinoma cells, positive control), and PBS control. Cells (2 × 10^6^) of each cell type were suspended in 200 *μ*L PBS and injected into the left forelimb armpit of nude mice. After 5 weeks, the mice were euthanized with CO_2_ overdose to harvest the implants. Slides were made and stained with haematoxylin and eosin dye to observe possible tumour growth.

### 2.11. Cell Attachment Experiment

The SHED-Bmi1-EGFP P40 attachment experiment on zein/gelatine nanofibre scaffolds was performed according to a previous study [38]. Briefly, the cells were seeded on zein/gelatine nanofibre scaffolds in a 24-well plate with a density of 5 × 10^4^ cells/cm^2^. Then, 2 days later, the adhesion and spread of the cells to the scaffolds were observed with an LSM-780 laser scanning confocal microscope (Carl Zeiss, Oberkochen, Germany) directly without any staining.

### 2.12. In Vivo Imaging Experiment

SHED-Bmi1-EGFP P40 were harvested using 0.25% trypsin-EDTA, and the cells suspended in PBS (4 × 10^6^/100 *μ*L) were injected subcutaneously into the backs of nude mice. The treated mice were observed with a Xenogen IVIS Spectrum (Caliper Life Sciences, USA) right after the cells were injected.

### 2.13. Statistics

All data are expressed as the mean ± standard error from at least three independent experiments. The SPSS 20.0 software package (SPSS Inc., Chicago, IL, USA) was used for the statistical tests. One-way ANOVA was used for comparison among groups. The quantitative statistics analysis of Western blot data used Student's *t*-test. In this study, *P* < 0.05 was considered to indicate statistical significance.

## 3. Results

### 3.1. Establishment of the Immortalized Cell Line SHED-Bmi1-EGFP

**Figure 1:**
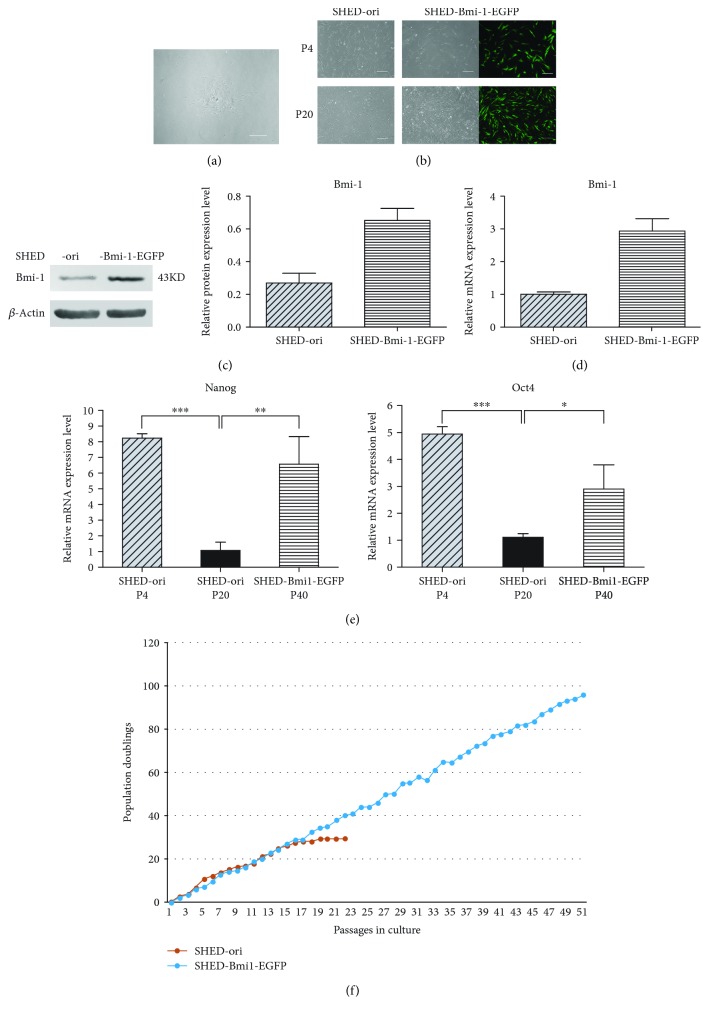
Establishment and verification of the immortalized cell line SHED-Bmi1-EGFP from primary SHED. (a) Representative image of colonies formed after 3 d of isolation. Scale bar, 200 *μ*m. (b) Representative images of nontransfected original cells (SHED-ori) and SHED-Bmi1-EGFP with different passages. Scale bar, 200 *μ*m. (c) Protein level of Bmi-1 in SHED-ori P4 and SHED-Bmi1-EGFP P40 was detected by Western blot. The relative protein expression levels were normalized to *β*-actin. (d) Expression levels of Bmi-1 were assayed with qRT-PCR. (e) Expression levels of Nanog and Oct4 were assayed with qRT-PCR. (f) Population doublings (PDLs) of SHED-ori and SHED-Bmi1-EGFP. Data are shown as the mean ± SDs of 3 separate experiments. ^∗^ *P* < 0.05, ^∗∗^ *P* < 0.01, and ^∗∗∗^ *P* < 0.001.

SHED were isolated from the dental pulp tissue of healthy human deciduous teeth and were mixed to decrease individual variation. After 3 days of isolation, the representative images of colonies were formed, and SHED were fibroblast-like cells (Figure 1(a)). The experiments to identify the fibroblast-like cells were also performed. The results confirmed that the cells we isolated and cultured from human deciduous teeth were mesenchymal stem cells (Figure S1). To establish the immortalized cell line SHED-Bmi1-EGFP, we constructed plasmid pMSCV-EGFP and infected SHED with EGFP lentivirus followed by Bmi-1 lentivirus. The morphologies of SHED and SHED-Bmi1-EGFP were analysed under a light microscope. SHED-Bmi1-EGFP, at passages 4 and 20, still maintained the shape of the nontransfected original cells (SHED-ori) at passage 4. Nevertheless, SHED-ori at passage 20 displayed senescent morphology and hardly continued to grow (Figure 1(b)).

### 3.2. Characterization of SHED-Bmi1-EGFP

The expression level of Bmi-1 in SHED-Bmi1-EGFP was evaluated with Western blot. Increased mRNA and protein expression of Bmi-1 was detected in SHED-Bmi1-EGFP at passage 40 compared with lower expression levels in SHED-ori (Figures 1(c) and 1(d)). This result confirmed the successful and stable expression of Bmi-1 during the passages. The expression levels of the stemness marker genes Nanog and Oct4 were detected with qRT-PCR. The results showed that the Nanog and Oct4 expression levels of SHED-Bmi1-EGFP P40 were both higher than those of SHED-ori P20 (Figure 1(e)). To evaluate the lifespan of SHED-Bmi1-EGFP, we tested the proliferative potential of SHED-Bmi1-EGFP. As shown in Figure 1(f), SHED-Bmi1-EGFP grew over 90 population doublings (PDLs), with stable propagation speed. However, SHED-ori entered crisis after approximately 25 PDLs.

### 3.3. Senescence Level and Proliferation Capacity of SHED-ori and SHED-Bmi1-EGFP

**Figure 2:**
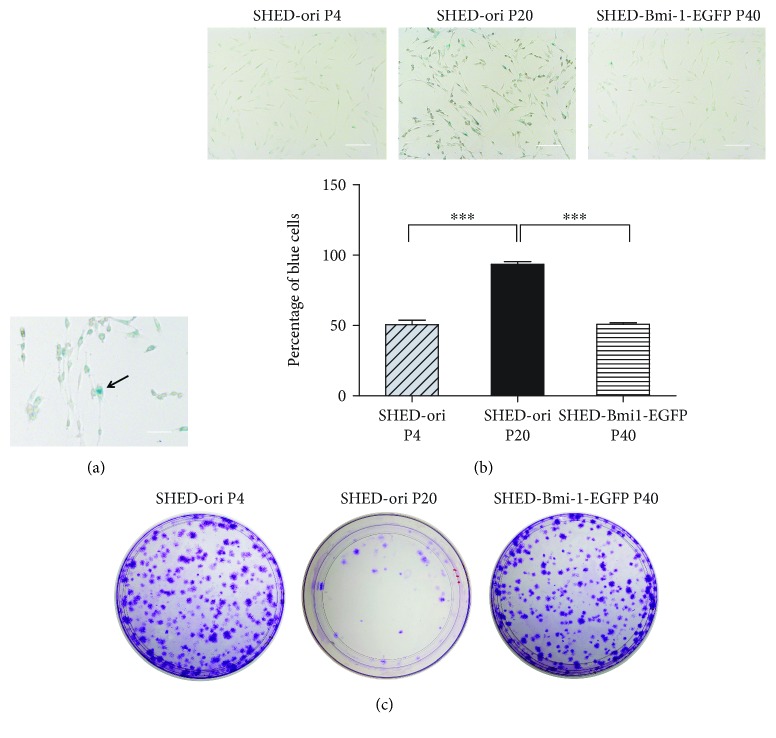
Senescence level and proliferation capacity of SHED-ori and SHED-Bmi1-EGFP. (a) Blue cells were counted as a measure of senescence. Scale bar, 100 *μ*m. (b) SHED-ori were stained with *β*-gal at P4 and P20. SHED-Bmi1-EGFP were stained at P40. Blue cells were counted as a measure of senescence. Scale bar, 200 *μ*m. (c) Colony formation assay was performed to measure the proliferation capacity of SHED-ori P4, SHED-ori P20, and SHED-Bmi1-GFP P40.

To evaluate the senescence level, a senescence-associated *β*-galactosidase assay was performed in SHED-ori and SHED-Bmi1-EGFP. There were numerous *β*-gal-positive cells in SHED-ori at the 20th passage, while few *β*-gal-positive cells were detected at the 40th passage of SHED-Bmi1-EGFP, which was identical to SHED-ori at the 4th passage (Figures 2(a) and 2(b)). Next, a colony formation assay was performed to measure the cell proliferation capacity. SHED-ori P4 and SHED-Bmi1-EGFP P40 clearly formed colonies after 14 days of culture, but SHED-ori P20 formed very few colonies (Figure 2(c)). Taken together, all the data indicated that SHED-Bmi1-EGFP bypassed senescence and retained high potential proliferation ability during long-term culture.

### 3.4. Multilineage Differentiation Assay of SHED-ori and SHED-Bmi1-EGFP

**Figure 3:**
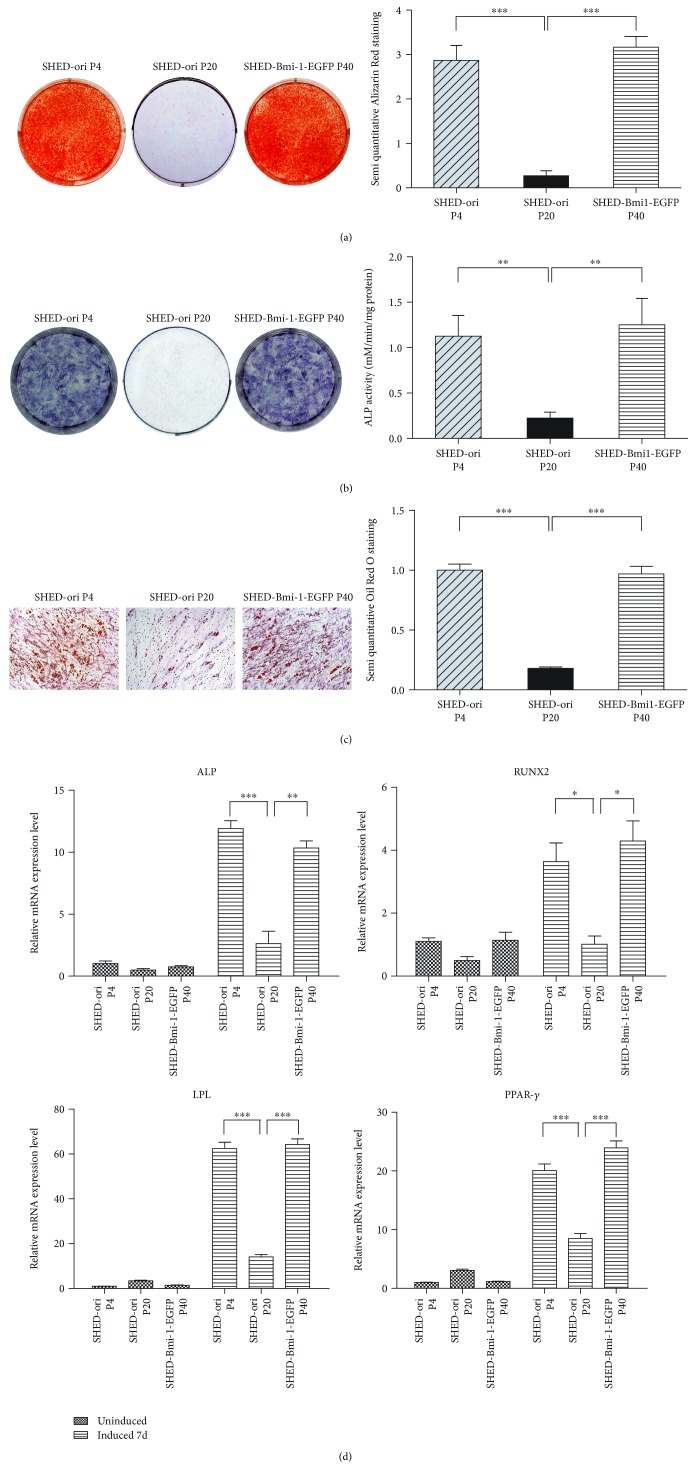
Multilineage differentiation assay of SHED-ori P4, SHED-ori P20, and SHED-Bmi1-EGFP P40. (a) Alizarin Red staining was performed to evaluate the osteogenic differentiation ability of the cells, and the semiquantitative results were calculated. (b) ALP staining and ALP activity assays were performed both in SHED-ori P4, SHED-ori P20, and SHED-Bmi1-EGFP P40. (c) Adipose droplets in cells were stained orange by Oil Red O after 21 days of adipogenic differentiation induction, and the semiquantitative results were calculated. (d) Osteogenic and adipogenic differentiation related genes were detected by qRT-PCR. Scale bar, 200 *μ*m. Data are shown as the mean ± SDs of 3 separate experiments. ^∗^ *P* < 0.05, ^∗∗^ *P* < 0.01, and ^∗∗∗^ *P* < 0.001.

To assess the multilineage differentiation potential of the immortalized cells, osteogenic and adipogenic differentiation induction was performed. Alizarin Red staining and the semiquantitative results revealed that mineralized nodules formed by SHED-ori P20 were significantly decreased compared to those formed by SHED-ori P4 and SHED-Bmi1-EGFP P40 (Figure 3(a)). The results of the alkaline phosphatase (ALP) staining and the ALP activity assay indicated that the ALP levels in SHED-ori P4 and SHED-Bmi1-EGFP P40 were significantly higher than those in SHED-ori P20 (Figure 3(b)). A similar outcome was shown in the Oil Red O staining assay. The adipose droplets in the SHED-ori P4 group and SHED-Bmi-EGFP P40 group were more and larger than those in the SHED-ori P20 group (Figure 3(c)). Osteogenic and adipogenic differentiation-related genes were detected by qRT-PCR, which showed that levels of the differentiation markers in SHED-Bmi1-EGFP P40 were all significantly higher when compared with those in SHED-ori P20 (Figure 3(d)). All these data demonstrated that SHED-Bmi1-EGFP P40 possessed similar multilineage differentiation capacity compared to SHED-ori P4.

### 3.5. Assessment of the Potential Tumourigenicity Ability of SHED-Bmi1-EGFP

**Figure 4:**
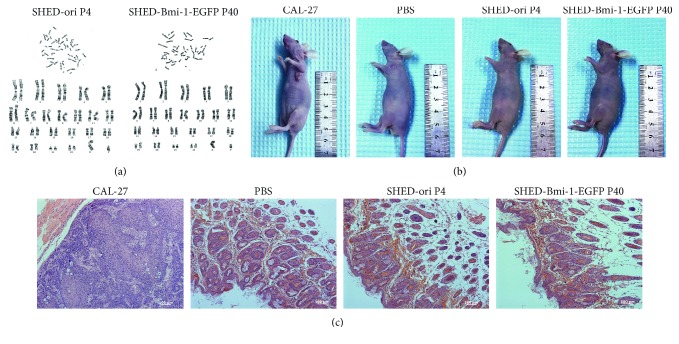
Assessment of potential tumourigenicity ability of SHED-Bmi1-EGFP. (a) Cytogenic analysis of immortalized cells. SHED-Bmi1-EGFP P40 showed no abnormality of karyotype. (b) Assessment of potential tumourigenicity of SHED-Bmi1-EGFP *in vivo*. In a total of 5 weeks after injections of cells into the left forelimb armpit, no tumour formation was seen in PBS, SHED-ori P4, or SHED-Bmi1-EGFP P40 group. (c) Tumour slides stained with haematoxylin and eosin dye. Positive control showed heteromorphism while PBS, SHED-ori P4, and SHED-Bmi1-EGFP P40 did not. Scale bar, 100 *μ*m.

Considering the potential risk of SHED-Bmi1-EGFP acquiring chromosomal changes due to genomic instability, we performed a cytogenic analysis on SHED-Bmi1-EGFP P40. As shown in Figure 4(a), SHED-Bmi1-EGFP P40 displayed 46 normal and sex chromosomal complements without polyploid mutations or chromosomal deletions, similar to SHED-ori P4. We performed a tumour-formation experiment in nude mice to evaluate the potential for tumourigenicity. SHED-ori P4, SHED-Bmi1-EGFP P40, and the positive control CAL-27 were inoculated with PBS. PBS without cells were the carrier-control group. After 5 weeks, no tumour formation was seen in the PBS, SHED-ori P4, or the SHED-Bmi1-EGFP P40 groups, but tumours larger than 1.0 cm^2^ were noticed in the CAL-27 group (Figure 4(b)). The analysis of HE-stained sections derived from tumours or relevant areas of inoculation showed that the CAL-27 cells formed squamous carcinoma with heteromorphism, but PBS, SHED-ori P4, or SHED-Bmi1-EGFP P40 did not show any signs of tumour growth (Figure 4(c)). All these data indicated that the immortalized SHED line we generated did not acquire the potential to form tumours in mice.

### 3.6. Potential Applications of SHED-Bmi1-EGFP

**Figure 5:**
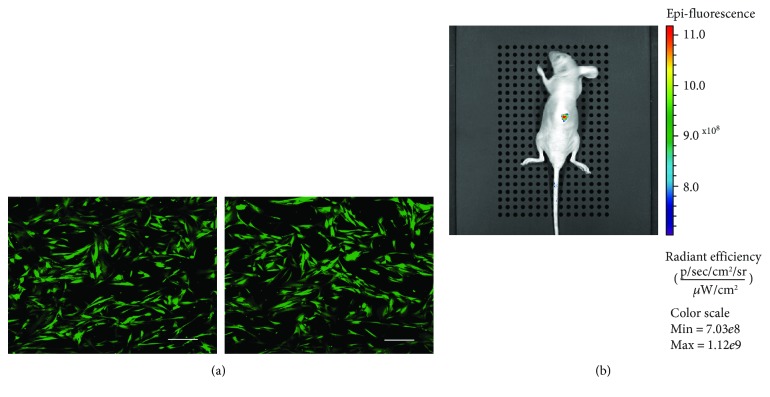
Potential applications of SHED-Bmi1-EGFP. (a) SHED-Bmi1-EGFP were seeded in zein/gelatine scaffolds and were observed with a confocal microscope. Scale bar, 200 *μ*m. (b) SHED-Bmi1-EGFP were injected on the backs of nude mice, and EGFP signalling was detected by the Xenogen IVIS Spectrum.

Since we want SHED-Bmi1-EGFP to be a tool in SHED research, we tried to find multiple uses for the cell line. EGFP has been widely used in scaffold assessments and *in vivo* studies. We seeded SHED-Bmi1-EGFP on the zein/gelatine scaffolds to observe adhesion as a part of cytocompatibility assessment (Figure 5(a)). The results can be seen intuitively without any traditional staining. Additionally, we injected the cells in the backs of nude mice and detected the EGFP signalling (Figure 5(b)). These data suggest that the cell line may be used extensively in SHED and tissue engineering research *in vitro* and *in vivo*.

## 4. Discussion

SHED has attracted much attention in recent years because of its many advantages. First, it is quite easy to access. Unlike other sources of mesenchymal stem cells, such as bone marrow mesenchymal stem cells and adipose-derived stem cells, SHED can be acquired during normal tooth development. This advantage increases its accessibility and leads to less trauma. Second, SHED is used widely in tissue engineering, especially in dental pulp regeneration as a special feature, because of its multilineage differentiation potential [12]. Stem cell-based bone regeneration is a research area with a long history associated with various kinds of stem cells, including SHED [39–44]. However, dental pulp regeneration is a research area that mainly focuses on two types of stem cells: DPSC [45–47] and SHED [10]. SHED has large potential in this research area. Third, SHED has a higher proliferation ability than DPSC or other sources of adult stem cells [14, 16–20], suggesting that SHED are more primordial. Based on these characteristics, we chose SHED to generate an immortalized cell line in this study. In doing so, we hope that it can be a practical tool for stem cell research.

During *in vitro* culture, SHED generally lose stemness [48], which suggests that one can establish an immortalized SHED cell line to act as a bank for long-term SHED research and generation dentistry. Many studies achieved immortalization with ectopic expression of Bmi-1 in various kinds of cells [26–28, 30]. In the cells we generated, a stable level of Bmi-1 was expressed (Figure 1(c)), and the lifespan successfully reached 50 passages and more than 90 PDLs (Figure 1(e)). A senescence-associated *β*-GAL staining and colony formation assay indicated that SHED-Bmi1-EGFP has similar senescence status and proliferation capability to SHED-ori P4 (Figure 2). Multilineage differentiation assays also showed the similarity of SHED-ori P4 and SHED-Bmi1-EGFP, indicating that the ectopic expression of Bmi-1 did not affect the stemness of differentiation (Figure 3). Much concern was raised about whether the immortalization would lead to aggressive tumours in animals. We conducted the *in vivo* tumourigenicity assay and acquired exciting results that the SHED-Bmi1-EGFP group did not form tumours after 5 weeks (Figures 4(b) and 4(c)), suggesting that the cells are not oncogenic. We will conduct further studies to assess the tumourigenicity up to 1 year after injection.

There has been research which reported that Bmi-1-mediated immortalized cell line of human placenta-derived mesenchymal cells showed extended proliferation after long-term growth arrest, while the cell line immortalized with hTERT plus Bmi-1 did not show such characteristic; besides, all the immortalized cells lost differentiation potential [49]. In another research, chromosomal instability in hMSCs immortalized with Bmi-1 and hTERT genes was compared [50]. Interestingly, it was found that the cells immortalized with the hTERT gene alone exhibited little change in karyotype, while chromosome numbers of the cells immortalized with Bmi-1 plus hTERT genes were unstable regarding chromosome numbers during long-term culture. There was a study used to establish an immortalized SHED cell line using ectopically stable expression of TERT [51]; here, we achieved it in a different way and proved our cell line SHED-Bmi1-EGFP could be passaged in long-term culture and maintained multiple differentiation capacity, indicating that unlike the results in previous studies, immortalization via overexpressing Bmi-1 in SHED did not affect its function as a tool cell line.

On the basis of immortalization, we marked the cells with EGFP to extend its potential application fields. Green fluorescent protein (GFP) reporter expression is widely used for stable labelling in live cells and tissues [52–54], after being originally derived from the bioluminescent jellyfish *Aequorea victoria*. The fluorescence can be seen and quantified by using noninvasive methods like a fluorescence microscope, confocal microscope, and flow cytometry. EGFP was generated by enhanced fluorescent signal, by labelling the target cells more explicitly and by adding to the range of applications [55]. In this study, we find some potential uses for SHED-Bmi1-EGFP, including the cytocompatibility assessment of biomaterial scaffolds as well as *in vivo* tracking (Figure 5).

Our data suggested that Bmi-1 can immortalize SHED without affecting its phenotype, senescence level, proliferation capacity, karyotype, and tumourigenicity ability. Previous studies suggested that the TERT immortalization increased the differentiation ability of mesenchymal stem cells [56], but we did not detect a significant difference of osteogenesis and adipogenesis between SHED-ori P4 and SHED-Bmi1-EGFP (Figure 3). It is widely known that mesenchymal stem cells also possess other features, such as immunomodulation and angiogenesis. It has been reported that overexpressing Bmi-1 in lymphocytes inhibits T cell activation thus playing an immunomodulative role in ovariectomy-induced bone loss [57]. Overexpression of Bmi-1 in bone cancer cells promotes proliferation and angiogenesis and increases apoptosis resistance induced by cisplatin via the nuclear factor-kappa B (NF-kappa B) signal pathway [58]. These researches demonstrate that overexpression of Bmi-1 may have an impact on the immunomodulatory as well as angiogenic properties, and whether SHED-Bmi1-EGFP acts differently from the original SHED on these two aspects deserves further research.

Stem cell-based tissue regenerative engineering has been an alternative therapy with great promise for many diseases. As SHED is a great potential source of seed cells, the cell line we conducted can play a critical role in many research aspects associated with tissue regenerative engineering, such as dental pulp regeneration, bone regeneration, and nerve regeneration because of its features. We believe there are more application ways of SHED-Bmi1-EGFP, which deserve further explorations; however, it is undeniable that direct application of SHED-Bmi1-EGFP in patients deserves meticulous and cautious considerations. On the one hand, although our data found that SHED-Bmi1-EGFP did not acquire the potential to form tumours in nude mice, it is still unsure whether it is tumourigenic in human. On the other hand, scaffolds and growth factors are two other important parts of tissue regenerative engineering besides stem cell. Whether the growth factors that have significant effects on original SHED would obtain the comparable achievements in SHED-Bmi1-EGFP and whether there exists a possibility that certain growth factors would play a distinct role in SHED-Bmi1-EGFP need to be further studied before the application in patients.

## 5. Conclusions

In this study, we established a lentivirus-mediated immortalized SHED cell line marked with EGFP that has great application potential. Our data demonstrated that the cell line stably expressed EGFP and could be passaged for a long time. We also showed that the immortalization did not affect the multipotency of SHED. We confirmed that the cells had no tumourigenicity. This study provides critical evidence that SHED-Bmi1-EGFP could be a tool for SHED research, without the need to collect SHED constantly from patients.

## Figures and Tables

**Table 1:** Primer sequences used in quantitative real-time reverse transcription polymerase chain reactions.

Gene target	Sequence
Bmi-1	Forward: 5′-TGGACTGACAAATGCTGGAGA-3′
	Reverse: 5′-GAAGATTGGTGGTTACCGCTG-3′
Nanog	Forward: 5′-AAGGCCTCAGCACCTACCTA-3′
	Reverse: 5′-TGCACCAGGTCTGAGTGTTC-3′
Oct4	Forward: 5′-GGCCACACGTAGGTTCTTGA-3′
	Reverse: 5′-GGCTGAATACCTTCCCAAATAGA-3′
ALP	Forward: 5′-TTCAAACCGAGATACAAGCACT-3′
	Reverse: 5′-GGGCCAGACCAAAGATAGAG-3′
RUNX2	Forward: 5′-TGGTTACTGTCATGGCGGGTA-3′
	Reverse: 5′-TCTCAGATCGTTGAACCTTGCTA-3′
LPL	Forward: 5′-TCATTCCCGGAGTAGCAGAGT-3′
	Reverse: 5′-GGCCACAAGTTTTGGCACC-3′
PPAR-*γ*	Forward: 5′-ATGGTGGACACGGAAAGCC-3′
	Reverse: 5′-CGATGGATTGCGAAATCTCTTGG-3′
GAPDH	Forward: 5′-AGGTCGGAGTCAACGGATTTG-3′
	Reverse: 5′-AGGCTGTTGTCATACTTCTCAT-3′

## Data Availability

The data used to support the findings of this study are included within the article.

## Supplementary Materials

Supplementary Materials Supplementary Figure 1: characterization of SHED. (A) The morphology of colony-forming SHED. (B) Representative images of a single colony-forming unit of SHED at 12 days. (C-D) Multiple differentiation ability of SHED. SHED demonstrated by Alizarin Red (C) and (D) Oil Red O staining under specific differentiation conditions for osteoblasts or adipocytes. (E) Cell surface markers of SHED were detected by flow cytometry. Scale bar: 200 *μ*m.
